# Schedule-dependent increased efficiency of pemetrexed-ionizing radiation combination therapy elicits a differential DNA damage response in lung cancer cells

**DOI:** 10.1186/s12935-016-0346-x

**Published:** 2016-09-02

**Authors:** Patrick Dorn, Colin Charles Tièche, Ren-Wang Peng, Laurène Froment, Ralph Alexander Schmid, Thomas Michael Marti

**Affiliations:** 1 Division of General Thoracic Surgery, Inselspital, Bern University Hospital, Murtenstrasse 50, 3008 Bern, Switzerland; 2 Department of Clinical Research, University of Bern, Bern, Switzerland

**Keywords:** Pemetrexed, Ionizing radiation, Chemoradiotherapy, Non-small cell lung cancer, DNA damage, Senescence

## Abstract

**Background:**

Lung cancer causes the most cancer deaths worldwide, thus there is a urgent need to develop new treatment options. Concurrent chemoradiotherapy has become a common strategy for the treatment of non-resectable solid tumors including non-small cell lung cancer. Pemetrexed is a folic acid antagonist that inhibits the synthesis of precursor nucleotides, whereas ionizing radiation induces DNA damage, the repair of which is dependent on sufficiently high nucleotide levels. In the clinical setting, the pemetrexed-ionizing radiation combination therapy is administered concomitantly. We hypothesized that prolonged pretreatment with pemetrexed could be beneficial, as prior depletion of nucleotide pools could sensitize cancer cells to subsequent irradiation.

**Methods:**

Non-small cell lung cancer A549 cells were treated with 1 µM pemetrexed for 72 h. In addition, cells were exposed to five gray ionizing radiation either 1, 48 or 71 h after the initiation of the pemetrexed treatment. Cell growth, senescence induction, cell cycle distribution and DNA damage marker accumulation were analysed at different time points during the treatment and the recovery phase.

**Results:**

Stand-alone treatments of five gray ionizing radiation and 1 µM pemetrexed resulted in an intermediate cell growth inhibition of A549 cells and were therefore applied as the combination regimen. Prolonged pemetrexed pretreatment for 71 h resulted in a significant S-phase accumulation. Irradiation and prolonged pemetrexed pretreatment maximally delayed long term cell growth. Additionally, senescence was augmented and recovery from treatment-induced DNA damage was most prominently delayed by prolonged pemetrexed pretreatment.

**Conclusions:**

Pretreatment with pemetrexed increases anticancer efficiency of pemetrexed-ionizing radiation combination therapy, which correlates with a persistence of treatment-induced DNA damage. Therefore, this study warrants further investigations to elucidate whether a similar adaptation to the standard treatment regimen could enhance the effectiveness of the non-small cell lung cancer clinical treatment regimen.

**Electronic supplementary material:**

The online version of this article (doi:10.1186/s12935-016-0346-x) contains supplementary material, which is available to authorized users.

## Background

Lung cancer is the leading cause of cancer-related mortality, resulting in over 1 million deaths worldwide each year. This is partly due to the difficulty in detecting the disease at early and more treatable time points, and a lack of effective treatment methods. Non-small cell lung cancer (NSCLC) accounts for approximately 85 % of all lung cancers, and approximately one-third of patients present with locally advanced NSCLC. For these patients, curative treatment is a challenge since the majority have unresectable bulky disease or extensive mediastinal lymphadenopathy resulting in a low 5-year survival rate (reviewed in [1]). For patients with locally advanced unresectable disease, concurrent chemoradiotherapy is considered the standard therapeutic approach (reviewed in [2]). However, the optimal chemotherapy regimen for use in conjunction with concurrent thoracic radiotherapy (RT) is still under debate. The combination of pemetrexed (multitargeted antifolate, MTA; commercial name ‘Alimta’) with cisplatin was recently recommended as the gold standard therapy for adenocarcinoma lung cancer patients with good performance status [3]. Two clinical trials evaluated single-agent MTA plus RT without consolidation therapy concluded that MTA can be given at full dosages during RT (reviewed in [2]). MTA is a folic acid antagonist inhibiting the synthesis of precursor purine and pyrimidine nucleotides that are required for DNA and RNA synthesis. MTA thereby interferes with the proliferation and survival of replicating cancer cells. Prolonged treatment with MTA induces replicative stress in the form of single stranded DNA, which, if not repaired, can lead to the formation of DNA double strand breaks (DSB) [4]. Ionizing radiation (IR) induces extensive base damage and creates DNA single strand breaks, resulting in DSB formation when two single strand nicks are present in complementary DNA strands within one helical turn (reviewed in [5]). DSBs are amongst the most cytotoxic DNA lesions, activating cell death response if unrepaired, and promoting genome instability if misrepaired (reviewed in [6]). DNA repair synthesis is a crucial step common to the various DSB repair mechanisms, and it has been proposed that the availability of nucleotide substrates for DNA repair synthesis may be a limiting factor for DSB repair [7]. Indeed, MTA pretreatment for up to 24 h prior to irradiation has been shown to enhance radiation-induced inactivation of lung carcinoma cells in vitro [8, 9]. We have recently shown that prolonged MTA pretreatment for 48 h augments persistence of cisplatin-induced DNA damage and eliminates resistant lung cancer cells (Tièche et al., manuscript in preparation). Thus in the present study, we hypothesized that prolonged pretreatment with pemetrexed could also be beneficial to radiation-induced therapy, as prior depletion of nucleotide pools could similarly sensitize cancer cells to subsequent irradiation.

In this study, we optimized MTA-IR anticancer treatment modality, and performed an in-depth molecular and cellular analysis to elucidate the molecular mechanisms underlying the observed benefit of sequential combination therapy. We demonstrated that prolonged MTA pretreatment improved the combination therapy’s efficiency. This effect correlated with the induction of persistent DNA damage and senescence initiation.

## Methods

### Cell culture and reagents

The NSCLC cell line A549 was cultured in Dulbecco’s’ modified Eagle’s medium nutrient mixture F-12 Ham (Cat. #D6421, Sigma-Aldrich, St. Louis, MO, USA), supplemented with 10 % fetal bovine serum (Cat. #10270-106; Life Technologies, Grand Island, NY, USA), 1 % penicillin/streptomycin solution (Cat. #P0781, Sigma-Aldrich) and 1 % l-glutamine (Cat. #25030-024, Sigma-Aldrich) at 37 °C in a humidified 5 % CO_2_ incubator. The cell line was previously DNA fingerprinted (Microsynth, Bern, Switzerland). Medium was changed every 3 days.

Pemetrexed/MTA (commercial name ‘ALIMTA’; Cat #VL7640) was purchased from Eli Lilly (Suisse) S.A. (Vernier/Geneva, Switzerland).

### Drug response and senescence associated β-galactosidase assay

To determine cell growth during the treatment and the initial recovery phase, 0.2 × 10^6^ cells were seeded into 150 mm × 20 mm tissue culture treated plates (Cat. #20151, SPL Life Sciences Co., Ltd, Korea). Starting at the day after seeding, i.e. day 0, cells from one plate per treatment were harvested using TrypLE (Cat. #12604021, Invitrogen, Grand Island, NY, USA). Cell titers were determined using a hemocytometer and trypan blue (Sigma-Aldrich) (final concentration 0.1 %) for dead cell exclusion. The cells were washed in phosphate-buffered saline and processed for analysis by flow cytometry as described below. To determine cell growth during the extended recovery period, cells were harvested at day 9 of the recovery period, reseeded at a 1:10 ratio into 150 mm × 20 mm plate. At day 13, cells were harvested and the titer was determined as described above, the subsequent flow cytometric analysis was performed as described below. Experiments were repeated independently three times.

Senescent cells were visualized by using the senescence associated β-galactosidase assay (Cat. #20151, Cell Signaling Technology, MA, USA). In detail, at day 6 after each treatment cells were fixed and stained overnight according to the manufacture’s protocol. An inverted light microscope (Eclipse TS100, Nikon Instruments Inc., Melville NA, USA) equipped with a 10× objective was used for visual quantification of senescent cells. Experiments were repeated independently three times.

### Flow cytometry

For analysis by flow cytometry, cells were harvested as described above. Subsequently, cells were washed with phosphate-buffered saline, pH 7.4, fixed and permeabilized with Cytofix/Cytoperm solution [Cat. #554714, BD Biosciences (San Jose, CA, USA)]. Staining with mouse Alexa Fluor 488 anti-γH2AX (Ser139) (Cat. #613406, Bio Legend, San Diego, CA, USA) antibody was performed in phosphate-buffered saline (Pharmacy, University Hospital Bern, BE, Switzerland) supplemented with 0.5 % saponin (Sigma-Aldrich) and 1 % bovine serum albumin (Sigma-Aldrich) on a rotating wheel (3 revolutions per minute) overnight at 4 °C. Subsequently, cells were treated with 100 µg/ml RNase A (Sigma-Aldrich) and DNA was stained simultaneously with 0.5 µg/ml 4′,6-diamidino-2-phenylindole (DAPI) (Sigma-Aldrich). Cell fluorescence was measured on a LSR2 upgraded flow cytometer (BD Biosciences) and analyzed using FlowJo V10 [Tree Star, Inc. (Ashland, OR, USA)]. Samples from the different time points were stored at 4 °C and flow cytometric analysis of all samples from one experiment was subsequently performed in parallel. Each analysis was accompanied by an untreated control. Buffer treated controls were used to set the gating threshold for γH2AX positivity to ~10 % of the total cell population as describe before [10].

### Statistical analysis

Data are presented as the mean ± standard deviation of at least three independent experiments if not stated differently. Data was analyzed using Excel software. Statistical differences were assessed using unpaired t test with Welch’s and P values <0.05 were considered significant.

## Results

### Optimization of the chemoradiotherapy treatment schedule increases anticancer efficacy

**Fig. 1:**
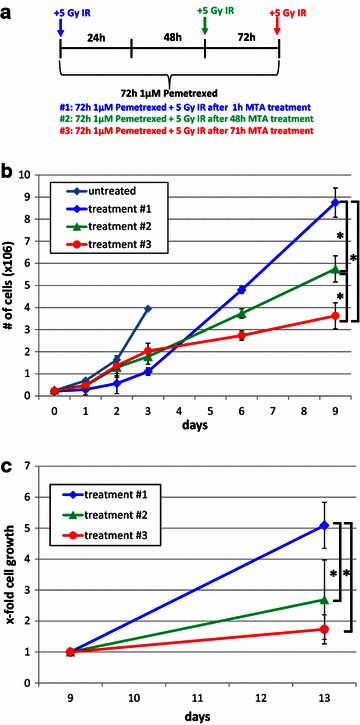
Optimization of the treatment schedule potentiates MTA-irradiation anticancer efficacy. **a** Schedule of the three tested treatment regimens differing in the duration of MTA pretreatment preceding irradiation. See text for details. **b** Growth curves of A549 cells during the treatment (0–3 days) and early recovery phase (up to 6 days post-treatment, e.g. day 9). **c** Cells exposed to the indicated treatment regimen were harvested at day 9 of the recovery phase, reseeded and cell numbers were determined at day 13 points. Data represent means of three independent experiments and *bars* indicate standard deviations. *P < 0.05

In order to optimize the treatment schedule of MTA plus irradiation, the concentration of MTA and level of exposure to irradiation that induced an intermediate level of growth inhibition was determined. Exposure to 5 Gy IR or treatment with 1 µM MTA resulted in an intermediate reduction of cell numbers (Additional file 1: Figure S1; Fig. 1b) and these conditions were therefore applied to the combination therapy. Three different treatment regimens were compared to determine whether the effectiveness of the MTA-IR combination therapy is dependent on the treatment schedule, including continuous MTA (1 μM) treatment for 72 h combined with exposure to 5 Gy IR at different time points (Fig. 1a). In detail, cells were irradiated 1, 48 or 71 h after the initiation of the 72 h MTA treatment (treatment #1, #2 or #3, respectively). The doubling time (day 0➙3) of untreated A549 cells was found to be approximately 22 h (Tièche et al., manuscript in preparation), which is in agreement with the information provided by the American Type Culture Collection. Compared to the untreated control, cell growth slightly decreased after 24 h MTA treatment alone (treatments #2 and #3, day 1) and further decreased after either 48 or 71 h MTA alone (treatment #3, day 2 or 3 respectively) (Fig. 1b). When the IR treatment was applied 1 h after the start of the MTA treatment (treatment #1), cell numbers did not increase considerably during the subsequent 24 h. In contrast, after pretreatment with MTA-for 48 h, cell numbers steadily increased during the 24 h following irradiation (treatment #2, day 2➙3). At the end of the treatment phase (day 4), the absolute cell counts were significantly lower after treatment #1 compared to treatment #2 and #3. However, during the extended recovery phase (day 6➙9), cell numbers steadily increased after treatment #1 whereas cell growth was significantly decreased after treatment #2 compared to treatment #1 and were further reduced after treatment #3 (Fig. 1b). In detail, during the recovery phase (day 6➙9) the doubling time of recovering cells after treatment #1 was 83 h whereas treatment #3 significantly prolonged the doubling time of the recovering cells (176 h) and with treatment #2 inducing an intermediate level of growth delay (116 h). To evaluate the growth capacity of the remaining cells, the residual cells were harvested at day 9 of the recovery phase and reseeded at low density. At day 13 of the recovery phase, cell numbers compared to treatment #1 were 1.9 and 2.9 times lower after treatments #2 and #3, respectively (Fig. 1c). Thus, treatment #3 reduced overall survival by a factor of ~7 compared to treatment #1 (fold difference in cell number at day 9 multiplied by day 13, i.e. 2.4 × 2.9). In other words, long-term cell growth was significantly reduced after extended pretreatment when compared to concomitant treatment.

### Prolonged MTA pretreatment and subsequent irradiation induces senescence

Prolonged cell cycle arrest after DNA damage induction results at the molecular level in DNA double strand break formation and at the cellular level in a terminal proliferation halt, i.e. senescence [11]. By permanently arresting proliferation of damaged cells, senescence serves as a barrier to cancer development.

**Fig. 2:**
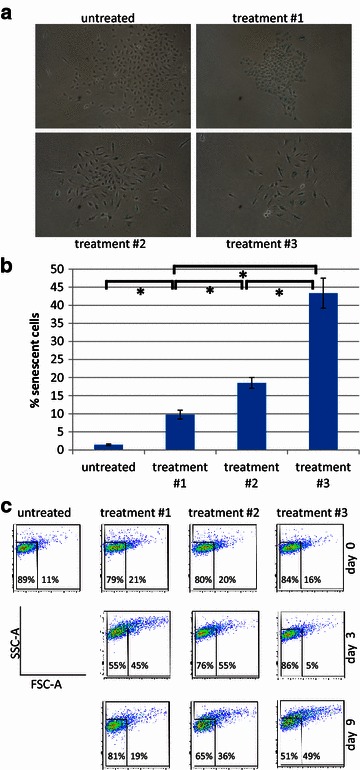
Prolonged MTA pretreatment augments irradiation-induced senescence in A549 cells. **a** Representative images of cells acquired by phase contrast-based microscopy at day 6. **b** Quantification of senescent cells based on increased β-galactosidase activity (see Fig. 2a). Data represent means of two independent experiments and *bars* indicate means and standard deviations. *P < 0.05. **c** Forward and side scatter analysis was performed by flow cytometry at the indicated time points. Data shown are the mean values and standard deviations of three independent experiments

Visual examination of the recovered cells after treatment #1 revealed that clones formed by small, cuboid cells could be distinguished from surrounding cells, which displayed morphologic changes that are associated with senescence, namely increased cell size and flattened shape (Fig. 2a; reviewed in [12]). The fraction of cells featuring a senescence phenotype was increased after treatment #2 and maximized after treatment #3 (Fig. 2a). Cells were stained to detect senescence-associated β-galactosidase (SA-β-Gal) activity. At day 6, the fraction of SA-β-Gal-positive cells (indicated by blue staining in Fig. 2a) was 4.5-fold higher after treatment #3 compared to treatment #1 (Fig. 2b, P > 0.05). Detectable by flow cytometry, increased forward (cell size) and side (cellular granularity) scatter intensity (F/S-high) is an additional feature associated with senescence (reviewed in [13]). Flow cytometric analysis at day 9 of the recovery phase revealed that the highest frequency of cells with a F/S-high phenotype was observed after treatment #3 (Fig. 2c). In detail, compared to the untreated control (13.3 %), the frequency of F/S-high cells was 1.5 and 3.3-fold higher after treatment #1 and treatment #3, respectively (19.8 and 43.2 %, P > 0.05), with treatment 2 inducing an intermediate level of senescence. Nevertheless, flow cytometric analysis at the end of the treatment phase (day 3) and during the early recovery phase (day 6) confirmed that for all three treatments, a fraction of the cells maintained a normal forward and side scatter intensity (F/S-low), indicating the presence of cells resistant to the tested treatment regimens (Additional file 2: Figure S2). However, compared to treatment #1, treatment #3 significantly decreased the fraction of resistant cells, e.g. F/S-low cells, indicating that extended MTA pretreatment can augment the anticancer activity of radiation therapy.

### Prolonged MTA pretreatment enhances S-phase accumulation prior to irradiation

**Fig. 3:**
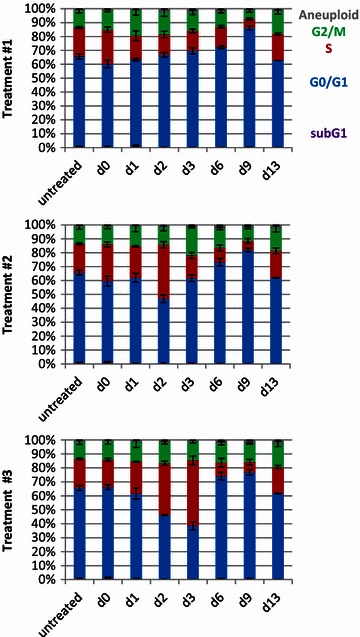
Prolonged MTA pretreatment exacerbates irradiation-induced cell cycle arrest and reduces the fraction of recovering cells. Flow cytometric analysis was performed at the indicated time points. Cell cycle analysis was performed as indicated in Additional file 3: Figure S3. Data shown are the mean values and standard deviations of three independent experiments

Terminal cell cycle arrest is a classic hallmark of senescence, and has been observed after treatment with chemotherapy (reviewed in [12]). Therefore, we monitored the cell cycle distribution of A549 cells during and after combined MTA-IR treatment (Fig. 3). MTA-alone treatment for 24 h did not result in a significantly changed cell cycle distribution (treatment #2 or 3, day 1). MTA-alone treatment for 48 h increased the fraction of cells in S-phase (treatment #3, day 2), which was even more pronounced after 72 h (treatment #3, day 3). Thus, at the start of the irradiation during treatment #1 (day 0), the cells mainly resided in the G1-phase of the cell cycle. In contrast, at the start of irradiation during treatment #2 (day 2), a significant fraction of the cells (37 %) was arrested in S-phase, which was further increased at the start of the irradiation during treatment #3 (day 3, 47 %).

Interestingly, when irradiation was preceded by MTA pretreatment for 48 h, the fraction of cells in S-phase decreased during the 24 h after irradiation (treatment #2, day 2 compared to day 3). An adverse effect of irradiation on the subsequent MTA-induced S-phase accumulation was also detectable after concurrent therapy. In detail, concurrent irradiation (treatment #1, day 0) completely abolished the MTA-induced S-phase accumulation at the end of the treatment phase (day 3, treatment #1 compared to treatment #3).

The cell cycle distribution of the recovering culture was normal during the early recovery phase (day 3➙6) after treatment #1. Since treatment #1 did not result in pronounced growth retardation, the cell culture became confluent during the later recovery phase (day 9) as also indicated by the accumulation in the G1-phase. Reseeding at low density revealed that the cell culture acquired a normal cell cycle distribution during the extended recovery phase, which was also detectable after treatment #2 and #3 (day 13). A sub-G1 DNA content is a hallmark of cells undergoing apoptosis. A very small fraction of sub-G1 cells (1.4 %) was observed 24 h after irradiation during treatment #1 (day 2) but not after treatment #2 (day 3) and also not 3 days after treatment #3 (day 6) indicating that the tested treatment regimen did not result in significant induction of apoptosis. In summary, pretreatment with MTA for a prolonged period enhanced S-phase accumulation prior to irradiation. However, this effect was transitory in the remaining, therapy-resistant fraction of the cells and cell growth returned to normal during the extended recovery phase.

### Prolonged MTA pretreatment results in persistent DNA damage accumulation

Induction of DNA breaks or DNA replication stress leads to the activation of the DNA damage response (DDR) (reviewed in [14]). During DDR, phosphorylation of histone variant H2AX (γH2AX) serves as a key mediator for the assembly of DNA repair proteins at the sites of DNA damage as well as for the activation of checkpoint proteins. Consequently, analysis of γH2AX is frequently used as a surrogate marker for DDR activation.

**Fig. 4:**
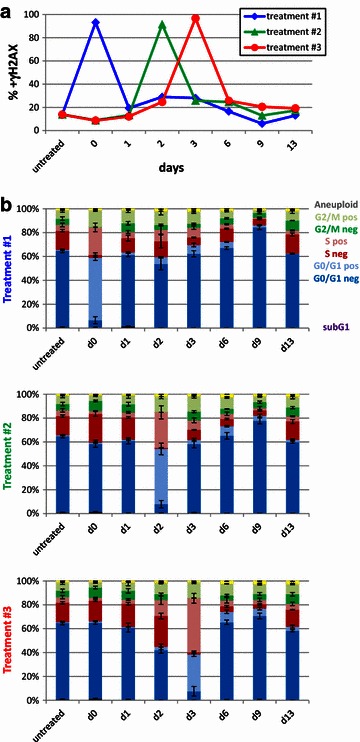
Prolonged MTA pretreatment enhances irradiation-induced accumulation of persistent DNA damage. Basal H2AX phosphorylation was set at ~10 % in untreated controls and used for normalization among experiments as described in the “Methods” section. **a** H2AX phosphorylation levels of the whole population were determined as described in Additional file 4: Figure S4. **b** Cell cycle phase-specific H2AX phosphorylation levels were determined by combining the γH2AX-positive and γH2AX-negative gates (Additional file 4: Figure S4) with the gates to determine the specific cell cycle phases (Additional file 3: Figure S3). Data shown are the mean values and standard deviations of three independent experiments. *neg* γH2AX-negative, *pos* γH2AX-positive

We have previously demonstrated that accumulation of persistent DNA damage leads to a cell cycle arrest and induction of senescence in A549 cells [15]. Thus, we determined the effect of the different treatment regimens on H2AX phosphorylation. Analysis of the total population revealed that irradiation with 5 Gy IR resulted in phosphorylation of H2AX in more than 90 % of the cells irrespective if MTA was administered concomitantly or as pretreatment (Fig. 4a). MTA-alone treatment for 24 h only marginally increased γH2AX levels in the total population (treatments #2 and 3, day 0➙1) (Fig. 4a). However, analysis of the cell cycle specific subpopulations revealed that MTA-alone treatment slightly increased γH2AX levels in S-phase and G2/M-phase cells whereas cells in the G1-phase were not affected (Fig. 4b). MTA treatment for 48 h (treatment #3, day 2) resulted in robust H2AX phosphorylation in a fraction of S-phase cells (34 %), which was also observed in the majority of cells in the G2/M-phase (66 %). After MTA-IR co-treatment, H2AX was rapidly phosphorylated in the majority of cells in all cell cycle phases (93 % of total population) (treatment #1, day 1, Fig. 4a). 24 h after irradiation, γH2AX phosphorylation returned to basal levels even in the presence of MTA co-treatment (treatment #1, day 0➙1). Similarly, H2AX phosphorylation was increased to nearly maximal levels in all phases of the cell cycle when irradiation was preceded by 48 or 71 h MTA pretreatment (91 and 97 % of total population, respectively) (treatment #2, day 2 and treatment #3, day 3, respectively). H2AX phosphorylation also returned to basal levels 24 h after irradiation during treatment #2 (treatment #2, day 2➙3). However, H2AX phosphorylation levels were still increased 3 and 6 days after termination of treatment #3 (day 6 and 9, respectively) compared to treatment #1 (25.7 and 20.5 % for treatment #3 compared to 16.5 and 6.0 % for treatment #1, respectively, P < 0.05) (Fig. 4a). During the recovery phase after treatment #3 (day 9), only a small fraction of cells in the G1-phase contained high levels of H2AX phosphorylation whereas persistent H2AX phosphorylation was detectable in the majority of cells in the G2/M-phase, which was absent after treatment #1 (Fig. 4b). In summary, prolonged MTA pretreatment (treatment #3) increased H2AX phosphorylation levels during the extended recovery phase.

In summary, the inhibitory effect of MTA-IR combination therapy on lung cancer cell growth can be further augmented by the optimization of the treatment schedule. Prolonged MTA pretreatment preceding irradiation reduces cell growth compared to concomitant treatment and increases the fraction of senescent cells. In addition, extended MTA-pretreatment induces a pronounced S-phase accumulation, which is abrogated by concomitant IR treatment. Finally, our investigations reveal that prolonged MTA pretreatment significantly delays recovery after DNA damage induction.

## Discussion

Concurrent chemoradiotherapy is considered the standard therapeutic approach for patients with locally advanced unresectable NSCLC. It is used for early treatment of micrometastatic disease and exploits the synergistic effect of the combination therapy in the shortest possible time frame (within several weeks), which explains its superiority compared to sequential therapy, i.e. several weeks of chemotherapy followed by radiation therapy (reviewed in [2]). The aim of the present study is the exploration of schedule-dependent effects within the concurrent MTA-IR combination therapy. The present in vitro study provides evidence to suggest that pretreatment with MTA prior to irradiation is significantly more efficient than concomitant treatment. These results are in agreement with previous studies showing that MTA pretreatment up to 24 h enhanced the radiation-induced inactivation of lung carcinoma cells in vitro [8, 9]. In a recent study, we demonstrated that prolonged MTA pretreatment for 48 h augments persistence of cisplatin-induced DNA damage and eliminates resistant lung cancer cells (Tièche et al., manuscript in preparation). Similarly, 24 h pretreatment with MTA sensitized A549 cells to a subsequent treatment with a histone deacetylase inhibitor and increased the survival-benefit of the combination treatment in a patient-derived lung cancer mouse xenograft model [16]. Thus, the present results are in agreement with the general consensus in the literature indicating that MTA pretreatment sensitizes NSCLC cells to a variety of cytotoxic drugs and treatments. The present study provides for the first time an in-depth analysis of the effects on NSCLC cells of the combined treatment of MTA and irradiation over an extended recovery period.

At end of the treatment phase (day 3), cell numbers were twofold lower after treatment #1 compared to treatment #3. Thus, a short-term analysis would therefore suggest treatment #1 to be the most efficient of the tested treatment regimens. However, treatment #3 was more effective over an extended recovery period. Tumor growth in animal models is usually monitored over periods of several weeks. Thus, it is tempting to speculate that tumor formation might be modelled in vitro more closely by the long-term growth analysis compared to a short-term study. However, further in vivo studies are needed to demonstrate the superior anticancer efficiency of pretreatment with MTA prior to ionizing radiation.

Although increased senescence was observed after treatment #1, as demonstrated by increased β-galactosidase activity, cell size and granularity, these senescence-associated effects were much more pronounced after treatment #3. Consistent with our findings of senescence induction, it has been shown that MTA-treated malignant mesothelioma cells also undergo accelerated senescence [17]. Senescent cancer cells can be cleared by the immune system, however the role of senescence in cancer progression is still controversial (reviewed in [12]). Senescence serves as a physiological barrier against tumor initiation and progression. On the other hand, senescent cancer cells might be able to overcome their dormant state, thus representing a dangerous potential for tumor relapse. It will therefore be crucial to utilize an immune competent animal model to determine the schedule-dependent anticancer efficiency of the combination therapy.

It is well established that the cell cycle status plays a critical role in the efficiency of combination chemotherapy. Unperturbed cells are maximally sensitive to treatment with IR during late G1/early S-phase and least sensitive during the peak of DNA synthesis in S-phase [18]. However, it has been shown that pretreatment of human colon carcinoma cells with the pemetrexed rendered not only G1-phase cells but also S-phase cells more sensitive to irradiation [19]. We showed that treatment with 1 µM MTA alone for 48 h resulted in the accumulation of cells in S-phase, which is in agreement with a previous study [20]. At the start of the irradiation, the fraction of cells in S-phase was highest in treatment #3 (day 3) compared to treatment #2 (day 2) and treatment #1 (day 1). Thus, we observed increased radiation sensitivity upon MTA-induced S-phase arrest, suggesting that it is not the S-phase status *per se* but is more likely to be the MTA-induced perturbation of DNA synthesis, which sensitizes cells to subsequent treatment with IR. Indeed, it has been reported that cells blocked at the G1/S-boundary remained sensitive to DNA damage induction after release of the block [21].

It was shown that the MTA-induced S-phase arrest in A549 cells relies on increased Cdk2/cyclin-A kinase activity, which itself was dependent on ERK-signaling pathway activity [20]. A subsequent study confirmed that the S-phase arrest upon MTA treatment in A549 cells is dependent on sustained Cdk2/cyclin-A activation although in this study, prolonged activation was found to be dependent on the PI3K/Akt signaling pathway [22]. Thus, the current literature indicates that the observed S-phase arrest upon MTA treatment is regulated by the complex interplay between various upstream signaling pathways. The dissection of this multifaceted signaling network will require an extensive analysis, ideally on the whole-genome transcriptome/(phospho-)proteome scale. Although beyond the scope of this study, this analysis might lead to the identification of targets and subsequently inhibitors with the potential to synergistically enhance the activity of the MTA-IR combination therapy.

Absolute cell numbers were significantly reduced 6 days after treatment #3 (day 9) compared to treatment #1, which was also observed after an extended recovery phase (day 13) (Fig. 1). However, comprehensive analysis revealed that at day 3 of the recovery phase (day 6) the cell cycle distribution of the remaining cells was similar to the untreated control (Fig. 3) indicating that at least a fraction of the recovering cells were proliferating. In summary, during the extended recovery period, a subpopulation of cells in all three treatment groups overcame cell cycle arrest and successfully completed mitosis, as also indicated by the increase of cells in the G1-phase at later recovery time points. Thus, this small fraction of cells is resistant to even the most efficient treatment regimen tested in this study, even though it is smaller in number than for other treatments.

Concomitant MTA-IR treatment of A549 cells led to a significant increase in H2AX phosphorylation, a marker for activation of the DNA damage response. Interestingly, similar γH2AX levels were reached when the radiation treatment was preceded by MTA pretreatment for 48 or 71 h, respectively. In other words, MTA pretreatment did not diminish H2AX phosphorylation upon IR-induced DNA damage formation. Furthermore, after MTA pretreatment for 48 h and concomitant MTA-IR treatment for 24 h, H2AX phosphorylation levels were only slightly increased compared to the untreated control. This suggests that the tested MTA treatment regimen (1 µM, 48 h) did not decreased cellular nucleotide levels to an extent that abolishes the repair of the IR-induced DNA damage. However, significantly higher γH2AX phosphorylation levels was observed during the recovery phase after treatment #3, in which irradiation was preceded by an extensive MTA pretreatment (3 days) indicating that prolonged MTA treatment might be required to augment the anticancer effect of the IR treatment. Indeed, we observed a significant increase in S-phase cells after 3 days compared to 2 days of MTA pretreatment, which was accompanied by increased H2AX phosphorylation in S-phase cells. It has been shown previously that nucleotide depletion leads to stalled replication forks, which progressively become inactivated and require two different RAD51-mediated pathways for restart and repair [23]. During S-phase, non-homologous end joining is repressed and DSBs are repaired by the RAD51-dependent homologous recombination pathway. Thus, we hypothesize that MTA-induced nucleotide depletion induces the sequestration of RAD51 to stalled or collapsed replication forks. Insufficient RAD51 levels limit the repair of IR-induced DNA DSBs by RAD51-dependent homologous recombination pathway resulting in the formation of complex DNA repair intermediates and the subsequent persistence of H2AX phosphorylation. However, further experiments will be required to elucidate the exact molecular mechanisms involved.

It has been previously demonstrated that treatment with 5-fluorouracil leads to the incorporation of 5-fluorouracil and uracil during S-phase, generating DNA repair-dependent, persistent DNA strand breaks during the successive G2/M-G1-phase, thereby interfering with the replication machinery in the subsequent S-phase [24]. The increased levels of H2AX phosphorylation at the extended recovery time points after treatment #3 therefore might be due to the persistence of complex DNA damage or repair intermediates. In this context, it has been shown previously that the persistence of H2AX phosphorylation 24 h after cisplatin treatment was associated with the loss of clonogenicity [25]. In summary, the analysis of H2AX phosphorylation levels have provided a first insight into the molecular mechanisms underlying the increased efficiency of the combination therapy after prolonged MTA pretreatment. Further studies will be necessary to elucidate the exact nature of the resulting DNA damage after prolonged MTA pretreatment.

Our study was restricted to the analysis of the cell line A549 featuring an activating mutation of the KRAS oncogene. In lung adenocarcinoma, oncogenic KRAS mutations are highly prevalent (~25 %) but therapy choices are very limited (reviewed in [3]) suggesting that our findings might be of relevance to advance treatment of a significant fraction of lung adenocarcinoma patients. However, further analysis of cell lines and primary cultures containing alternative mutational signatures will be necessary to evaluate if these findings are also of relevance for different lung cancer subsets.

MTA is administered as a daily 10-min infusion, which results in a relative rapid clearance from the body. However, MTA is efficiently converted intracellularly to an active polyglutamate form. Thus, although MTA blood levels decline rapidly, active MTA polyglutamate derivatives are sustained in tumor cells explaining the clinical efficiency of the initial phase I trials with single-agent MTA therapy, which was administered on day 1 every 21 days for up to six cycles (reviewed in [26]). The present study reveals that prolonged MTA pretreatment increases the efficiency of the combination MTA-IR therapy in vitro. Hence, it is tempting to speculate that a delay of the irradiation might also increase the efficiency of the MTA-IR combination therapy in the clinical setting.

## Conclusions

The present study has revealed that the efficiency of the MTA-IR combination therapy can be augmented in vitro by modifying the treatment schedule to include prolonged MTA pretreatment. The increased efficiency of this treatment can be attributed to the induction of persistent DNA damage, which in turn results in increased senescence initiation. Therefore, our study warrants further experiments to elucidate whether an optimization of the standard therapy schedule might also potentiate the current MTA-IR combination treatment regimen in vivo.

## Additional files

**Additional file 1: Figure S1.** Growth curves of A549 cells over time after exposure to ionizing radiation at the indicated intensities.

**Additional file 2: Figure S2.** Flow cytometric analysis of forward (cell size) and side (cellular granularity) scatter intensity as an alternative readout for senescence. Approximately 10 % of the cells of the untreated controls were placed in the F/S-high compartment and used as normalization standard as described in the “Methods” section. Forward and side scatter analysis by flow cytometry (without reseeding) at the indicated time points during the treatment and recovery phase. Shown are representative images of three independent experiments.

**Additional file 3: Figure S3.** Applied strategy to determine cell cycle phases by flow cytometry. Flow cytometric analysis was performed at the indicated time points. Gates set to determine the cell cycle distribution are indicated has horizontal bars. Indicated are days during treatment and the recovery phase. Data shown are representative of three independent experiments.

**Additional file 4: Figure S4.** Applied strategy to determine H2AX phosphorylation levels by flow cytometry. A 10 % threshold for basal H2AX phosphorylation levels was applied as indicated in the “Methods” section. Indicated are days during treatment and the recovery phase. Data shown are representative of three independent experiments.

## Abbreviations

NSCLC: non-small cell lung cancer
RT: radiotherapy
MTA: multitargeted antifolate, commercial name ‘Alimta’
DSB: DNA double strand break
IR: ionizing radiation
SA-β-Gal: senescence-associated β-galactosidase
DDR: DNA damage response
γH2AX: phosphorylation of histone variant H2AX
