# Supplementary figures and images for: Schedule-dependent increased efficiency of pemetrexed-ionizing radiation combination therapy elicits a differential DNA damage response in lung cancer cells
Source: Cancer Cell Int. 2016 Sep 2;16:66. doi: 10.1186/s12935-016-0346-x (PMC5010745; doi:10.1186/s12935-016-0346-x)

## Slide 1
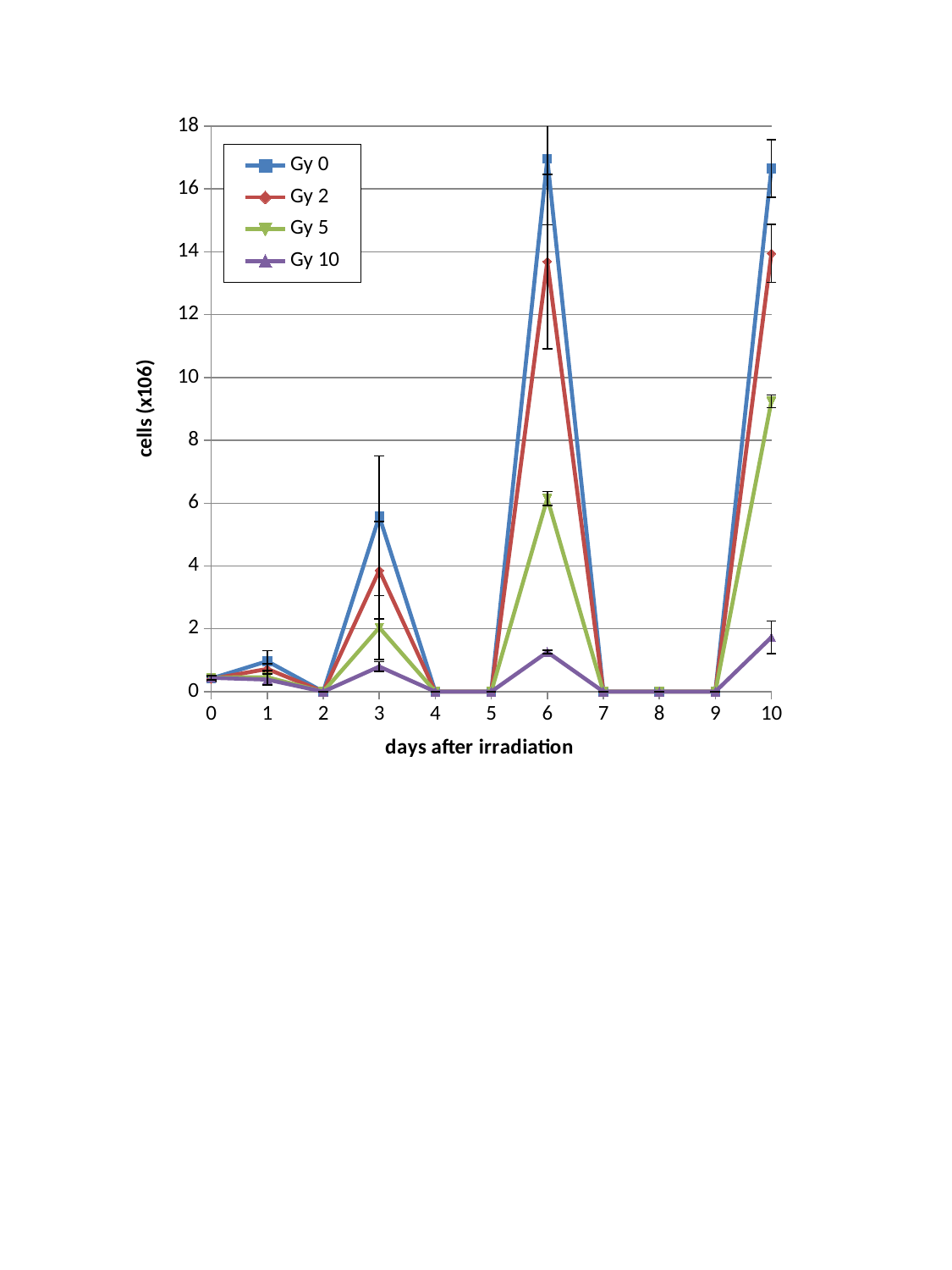

## Slide 2
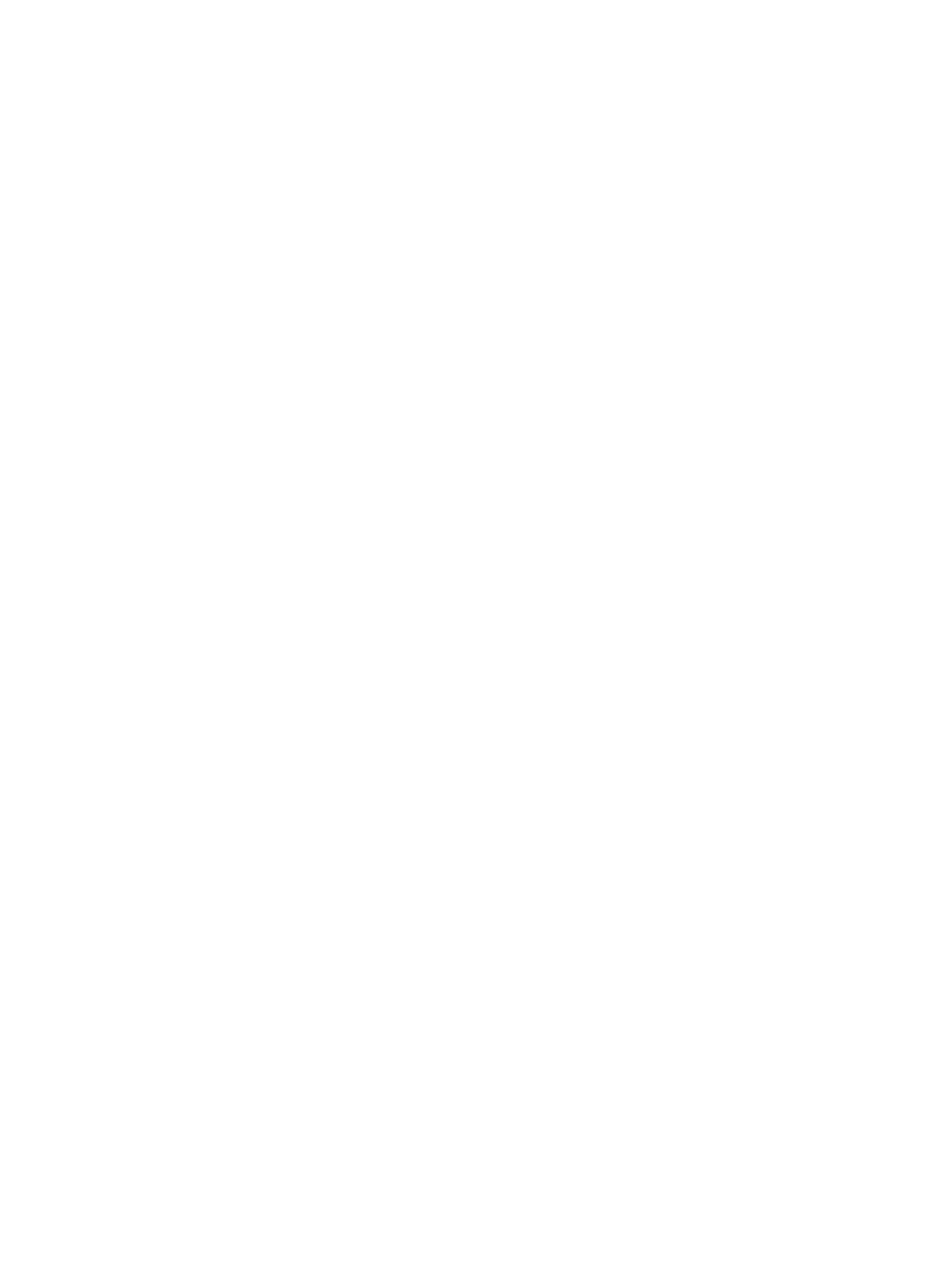

## Slide 3
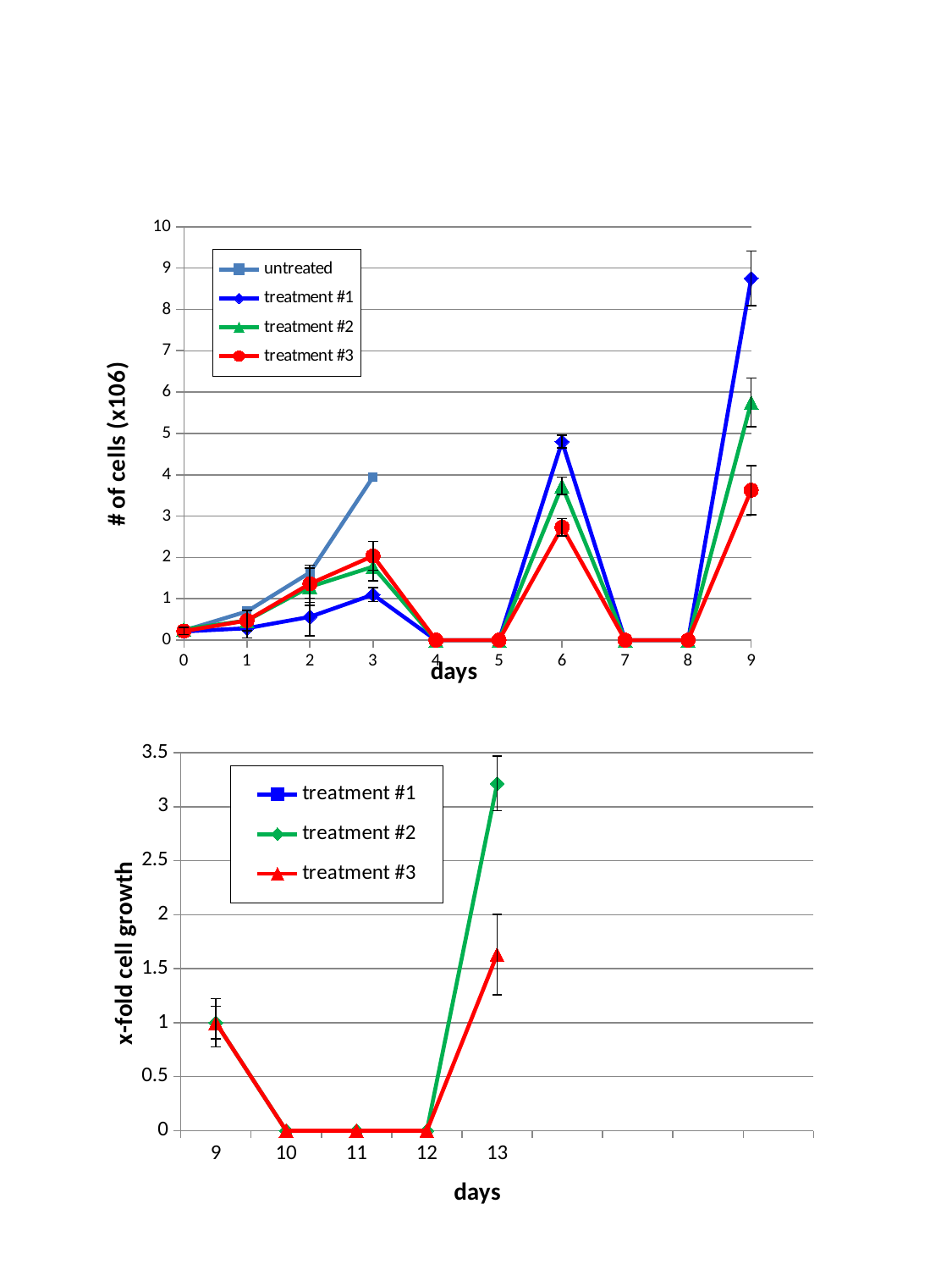

## Slide 4
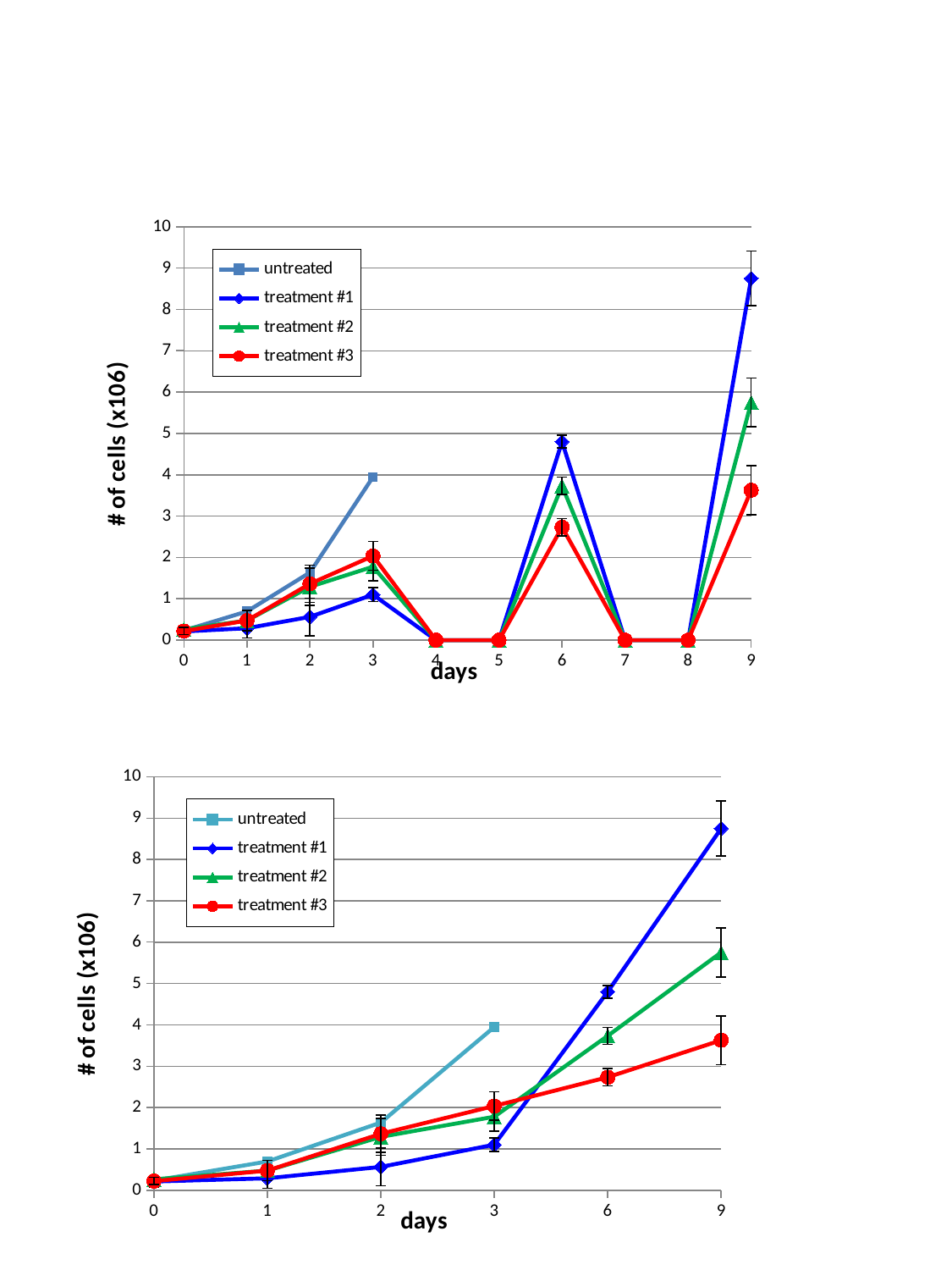

Supplement: Supplementary file 1 — Additional file 1: Figure S1. Growth curves of A549 cells over time after exposure to ionizing radiation at the indicated intensities. [file 12935_2016_346_MOESM1_ESM.pptx]

## Slide 1
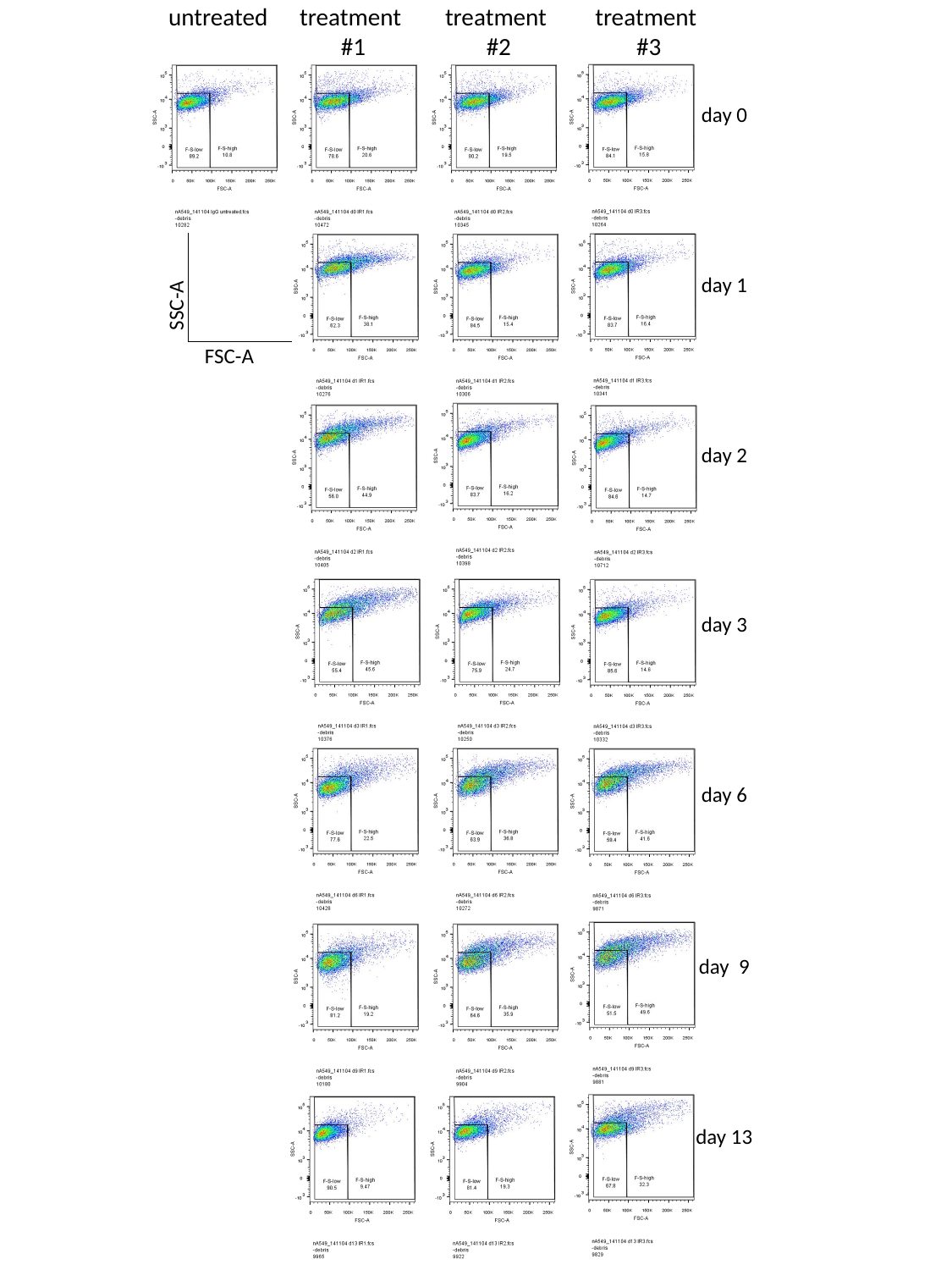

untreated
treatment
#1
treatment
#2
treatment
#3
day 0
SSC-A
day 1
FSC-A
day 2
day 3
day 6
day 9
day 13

Supplement: Supplementary file 2 — Additional file 2: Figure S2. Flow cytometric analysis of forward (cell size) and side (cellular granularity) scatter intensity as an alternative readout for senescence. Approximately 10 % of the cells of the untreated controls were placed in the F/S-high compartment and used as normalization standard as described in the “Methods” section. Forward and side scatter analysis by flow cytometry (without reseeding) at the indicated time points during the treatment and recovery phase. Shown are representative images of three independent experiments. [file 12935_2016_346_MOESM2_ESM.pptx]

## Slide 1
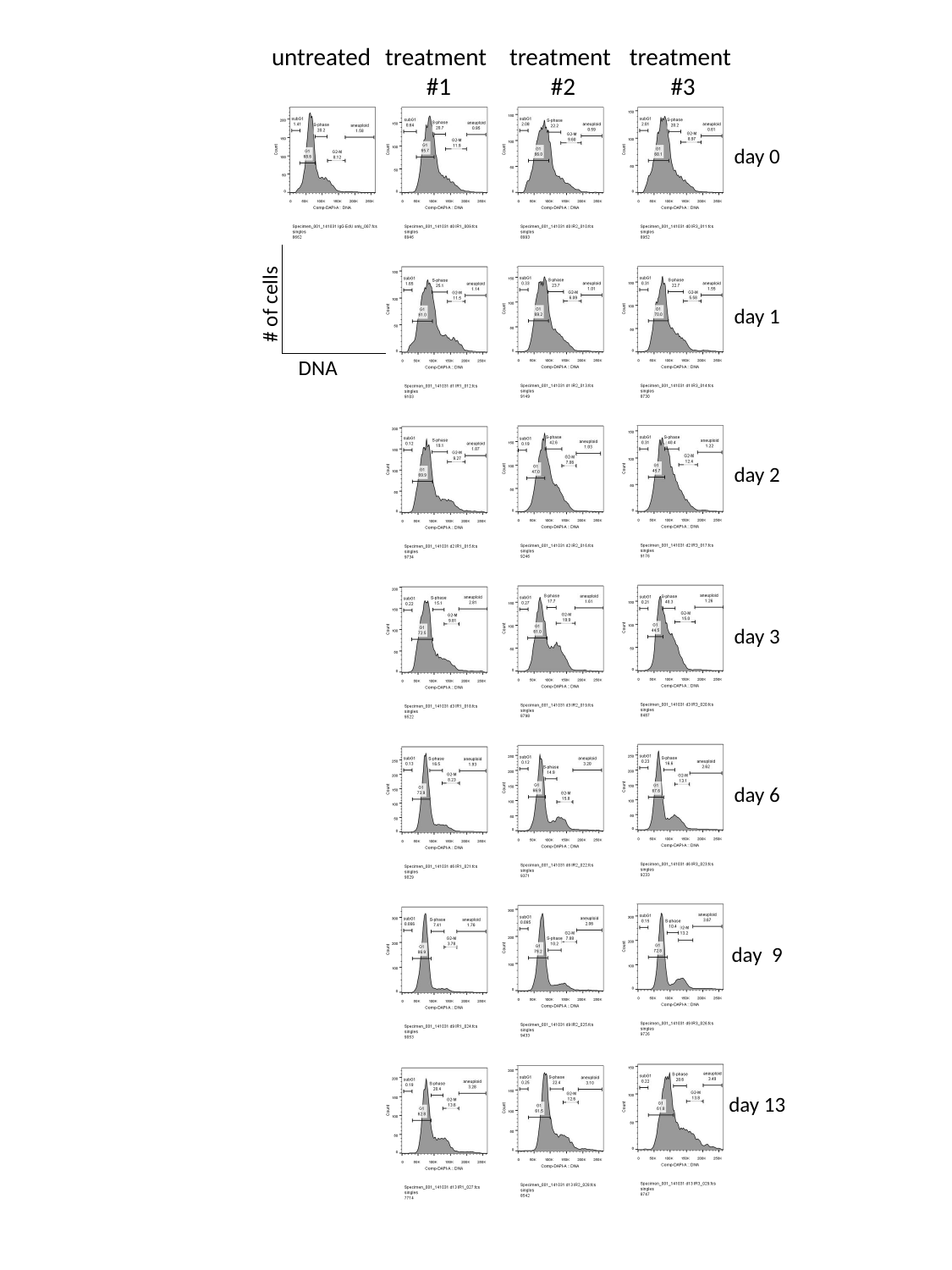

untreated
treatment
#1
treatment
#2
treatment
#3
day 0
# of cells
day 1
DNA
day 2
day 3
day 6
day 9
day 13

Supplement: Supplementary file 3 — Additional file 3: Figure S3. Applied strategy to determine cell cycle phases by flow cytometry. Flow cytometric analysis was performed at the indicated time points. Gates set to determine the cell cycle distribution are indicated has horizontal bars. Indicated are days during treatment and the recovery phase. Data shown are representative of three independent experiments. [file 12935_2016_346_MOESM3_ESM.pptx]

## Slide 1
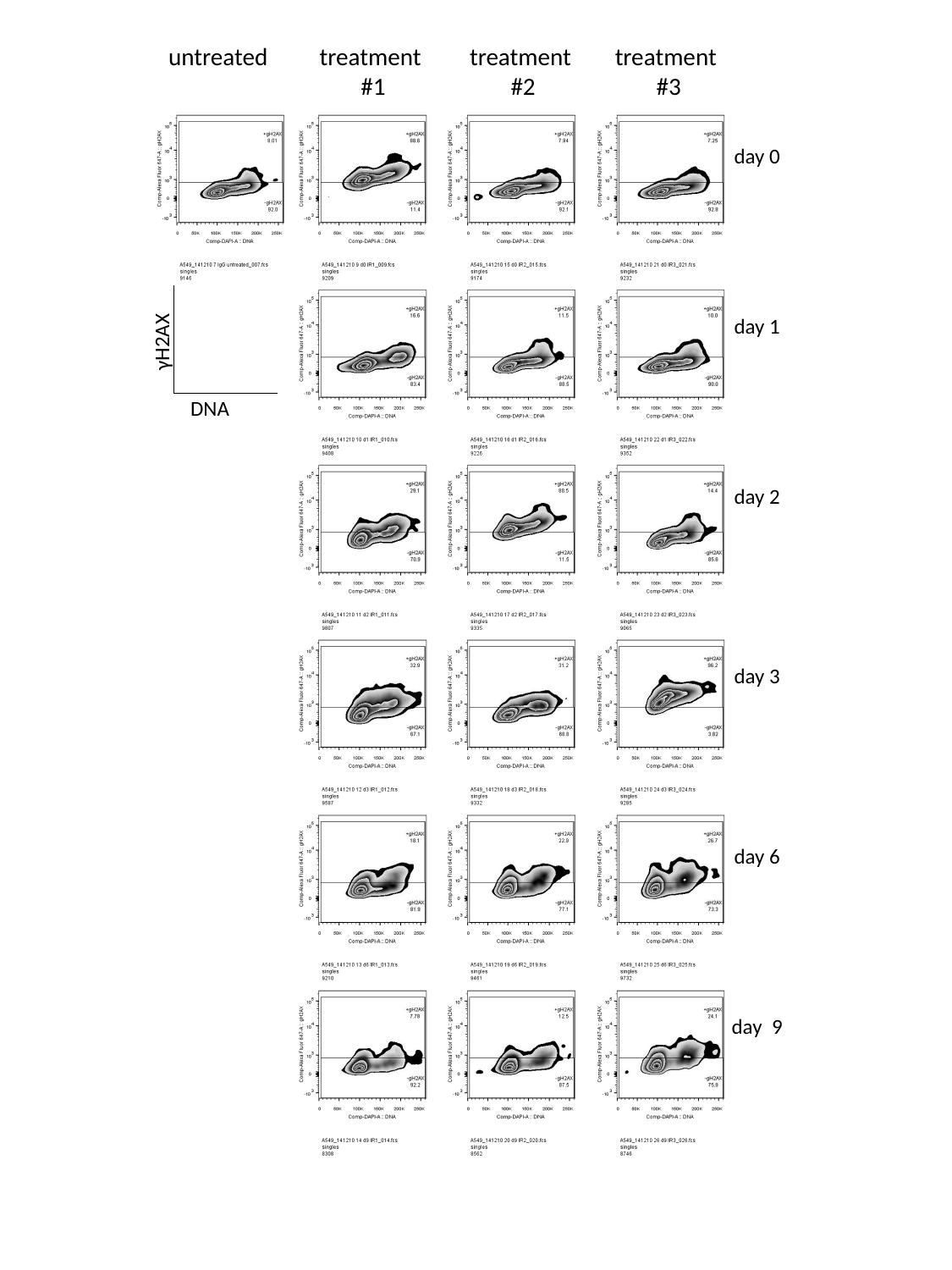

untreated
treatment
#1
treatment
#2
treatment
#3
day 0
day 1
γH2AX
DNA
day 2
day 3
day 6
day 9

Supplement: Supplementary file 4 — Additional file 4: Figure S4. Applied strategy to determine H2AX phosphorylation levels by flow cytometry. A 10 % threshold for basal H2AX phosphorylation levels was applied as indicated in the “Methods” section. Indicated are days during treatment and the recovery phase. Data shown are representative of three independent experiments. [file 12935_2016_346_MOESM4_ESM.pptx]
